# Associations between resting heart rate and cognitive decline in Chinese oldest old individuals: a longitudinal cohort study

**DOI:** 10.1186/s12877-023-04600-y

**Published:** 2024-01-04

**Authors:** Zhaoyin Ma, Yanlin Qu, Haibo Ma, Yuanyuan Zhang, Min Wang, Nana Huang, Xiaohong Li

**Affiliations:** 1 Department of Neurology, Jinan Central Hospital, Shandong University, Jinan, Shandong People’s Republic of China; 2 Medical Integration and Practice Center, Cheeloo College of Medicine, Shandong University, Jinan, Shandong People’s Republic of China; 3 Department of Ophthalmology, Shanghai General Hospital, Shanghai Jiao Tong University School of Medicine, Shanghai, China; 4 National Clinical Research Center for Eye Diseases, Shanghai, China; 5 Department of Neurology, Shandong Provincial Third Hospital, Shandong University, Jinan, Shandong People’s Republic of China; 6 Department of Neurology, Central Hospital Affiliated to Shandong First Medical University, Jinan, Shandong People’s Republic of China; 7 Medical Integration and Practice Center, Jinan Central Hospital, Shandong University, Jinan, Shandong People’s Republic of China

**Keywords:** Rest Heart Rate, Cognitive decline, Physical activity, The oldest old individuals, Longitudinal studies

## Abstract

**Background:**

The trajectories of cognitive function in the oldest old individuals is unclear, and the relationship between resting heart rate (RHR) and cognitive decline is controversial.

**Methods:**

3300 participants who had cognitive function repeatedly measured 4 ~ 8 times were included, and latent class growth mixed models were used to identified the cognitive function trajectories. Cognitive decline was defined by the trajectory shapes, considering level and slope. After excluding individuals with sinus rhythm abnormal, 3109 subjects were remained and were divided into five groups by their RHR. Logistic regression models were used to estimate the relationship between RHR and cognitive decline.

**Results:**

Three distinct cognitive function trajectory groups were identified: high-stable (n = 1226), medium-decreasing (n = 1526), and rapid-decreasing (n = 357). Individuals of medium/rapid-decreasing group were defined as cognitive decline. Adjusting for covariates, the odds ratios (95% confidence intervals) of RHR sub-groups were 1.19 (0.69, 2.05), 1.27 (1.03, 1.56), 1.30 (1.01, 1.67) and 1.62 (1.07, 2.47) for those RHR < 60 bpm, 70 ~ 79 bpm, 80 ~ 89 bpm and > 90 bpm respectively, compared with those RHR 60 ~ 69 bpm. The interaction effect between RHR and physical activity (PA) on cognitive decline was found, and stratification analysis was presented that higher RHR would only show risk effects on cognitive decline in those with physical inactivity (*P* < 0.05 for all).

**Conclusions:**

Our study demonstrates RHR more than 70 bpm present significant risk effect on cognitive decline, and this relationship is modified by PA. Elder population with physical inactivity and higher RHR should be paid more attention to prevent cognitive decline.

**Supplementary Information:**

The online version contains supplementary material available at 10.1186/s12877-023-04600-y.

## Introduction

Prevalence of dementia has greatly increased for decades, causing a severe health problem [1–3]. Researchers have demonstrated dementia would severely affect the quality of life, especially the oldest old individuals [3]. However, no cure for dementia is effective, currently. Identifying sub-group with cognitive decline steeply, and exploring the modifiable risk factors for cognitive decline would be the best way to delay the onset of dementia [4, 5]. Previous studies have been illustrated that cardiovascular diseases (CVDs) are significantly associated with the cognitive decline, because of their common risk factors [6–8]. Managing the risk factors of CVDs may delay the process of cognitive decline [6].

The associations between CVDs’ risk factors and cognitive decline have been well-established, such as body mass index (BMI) [7–11]. However, as an important risk factor of CVDs, the relationship of rest heart rate (RHR) and cognitive decline was still unclear. A few longitudinal studies have reported that higher RHR was associated with cognitive decline [6, 12, 13]. While these results were not proved by other researches [14, 15], leaving the relationship between RHR and cognition still controversial. Besides the different results, these studies usually defined cognitive decline by limited measurements, ignoring the longitudinal changing pattern in life-course [6, 12–15]. Some individuals with steep trend of decline may be misclassified because of the short duration of follow-up. We hypothesized that there are different patterns of cognitive trajectories in the oldest old people in China, and that different patterns of cognitive trajectories are closely related to RHR.

Using data from the Chinese Longitudinal Healthy Longevity Survey (CLHLS), this longitudinal research aims to identify cognitive function trajectories in the oldest old individuals (75 ~ 100 years), estimate the relationship between RHR and cognitive function trajectory groups.

## Subjects and methods

### Study cohort

As an ongoing cohort, Chinese Longitudinal Healthy Longevity Survey (CLHLS) was implemented by the Center for Healthy Aging and Development Studies at Peking University and the Center for the Study of Aging and Human Development at Duke University, jointly [16, 17]. Since 1998, comprehensive information from elderly individuals were collected, and seven follow-up waves of data were collected in 2000, 2002, 2005, 2008, 2011, 2014 and 2018. CLHLS randomly selected half of the total number of counties and cities in 22 out of 31 provinces in mainland China, conducted covered approximately 85% of the total population of China [16, 17].

In this current study, individuals with age > 100 or age < 75 (N = 15,206), missing data in cognitive function tests (N = 109), and with less than four follow-up visits (N = 38,334) were excluded. Finally, 3300 subjects (1487 males, ages 75 to 94 years at baseline), with 4–8 times examinations data, were included in this study to identify the trajectory of cognitive function. The mean follow-up duration was 10.1 years (sd = 2.4 years). Among these 3300 individuals, 3109 subjects (1401 males, mean age = 81.5 years) whose self-reported sinus rhythm were normal at baseline formed the current longitudinal study cohort.

The CLHLS study was approved by the Research Ethics Committee of Peking University (IRB00001052-13074), and all participants or their proxy respondents provided written informed consent.

### Exposure

Using standard electronic sphygmomanometer, RHR was measured after an hour rest at each follow-up in a sitting position. The peak pulse wave was recorded by the sphygmomanometer automatically during the measurement duration, and the RHR was calculated according to the number of pulse waves [18]. Participants were divided into 5 RHR categories, labeled as < 60 beats per minute (bpm), 60 ~ 69 bpm, 70 ~ 79 bpm, 80 ~ 89 bpm, and ≥ 90 bpm, and the 60 ~ 69 bpm group was defined as the reference group similar with the previous research [6].

### Covariates

In this current study, sociodemographic characteristics, health behaviors and health status at baseline were adjusted in the model. These covariates were selected a priori as potential confounders based on the literature [19, 20]. The sociodemographic characteristics were age (continuous), gender, education level (no schooling/some schooling), residence (city/town/village), marital status (married/other) and cohabitant (family/solitary/live in institution). Health behaviors were current smoking status (never or past/current), current alcohol drinking (yes/no), physical activity (PA) at present (yes/no), fruit intake (yes/no) and vegetable intake (yes/no). Health status was common chronic diseases (self-reported hypertension, diabetes, cardiopathy, and stroke) and body weight (continuous). In addition, a cohort identifier was established to indicate which cohort the participants were from.

### Outcome

Cognitive function was measured by the Chinese version of the Mini-Mental State Examination (MMSE) during each survey. The validity and reliability of the Chinese MMSE has been verified [16, 17]. Based on the literature [21], we treated responses of “unable to answer” as “wrong”. The MMSE score ranged from 0 to 30, and a higher score indicated better cognitive function. In order to identify the trajectory groups of cognitive function, a normalising transformation was applied to MMSE values, converting MMSE from a 0–30 scale to nMMSE on a 0–100 scale to deal with curvilinearity [22].

### Statistical methods

The latent class growth mixture model (LCGMM) was used to identify different trajectory patterns of MMSE [23]. The latent class trajectory of MMSE was specified as a function of age (centered to 86 years, the mean age of the cohort). Multiple LCGMMs with different trajectory shapes including linear and nonlinear parameters were tested [24–26]. Repeated trajectory analyses were performed to identify the latent classes by changing the number of groups from 2 to 4, with the same starting values calculated from the 1-group model. The class memberships of subjects were determined by a latent discrete random variable, and its probability is described using a multinomial logistic model according to covariates. We chose the best-fitting model for MMSE trajectory according to the BIC criterion while ensuring that each group has an acceptable proportion of the population and posterior probability.

Considering the changing patterns of MMSE, subjects were divided into cognitive decline group and normal group. Logistic regression models were used to explore the associations between the resting heart rate (RHR) and cognitive decline, adjusted for baseline age, gender, residence, education level, marital status, cohabitant, smoking and alcohol drinking status, fruit and vegetable intake, self-report hypertension, self-report diabetes, self-report stroke, self-report heart disease, body weight, and PA. Moreover, the interaction effect between RHR and PA was estimated by adding an interaction term in logistic regression model. Stratification analysis was used to explore whether the RHR performed different effects on cognitive decline by the PA status.

Variables were described using mean (sd), median [interquartile range] and n (%), as appropriate. All analyses were preformed using R version 4.0.4. Hypothesis tests were 2-sided, and *P* < 0.05 was considered statistically significant.

## Results

Supplement Table S1 summarizes LCGMM model fitting process of the MMSE trajectory. We fitted models from one class to four classes of linear, quadratic and cubic curves. According to statistical criteria above, a model of quadratic parameters with three classes was chosen as the best-fitting model. Supplement Table S2 presents parameter estimates for the best-fitting 3-class quadratic latent class growth mixed model. All the parameters were statistically significant (*P* < 0.05 for all).

**Fig. 1:**
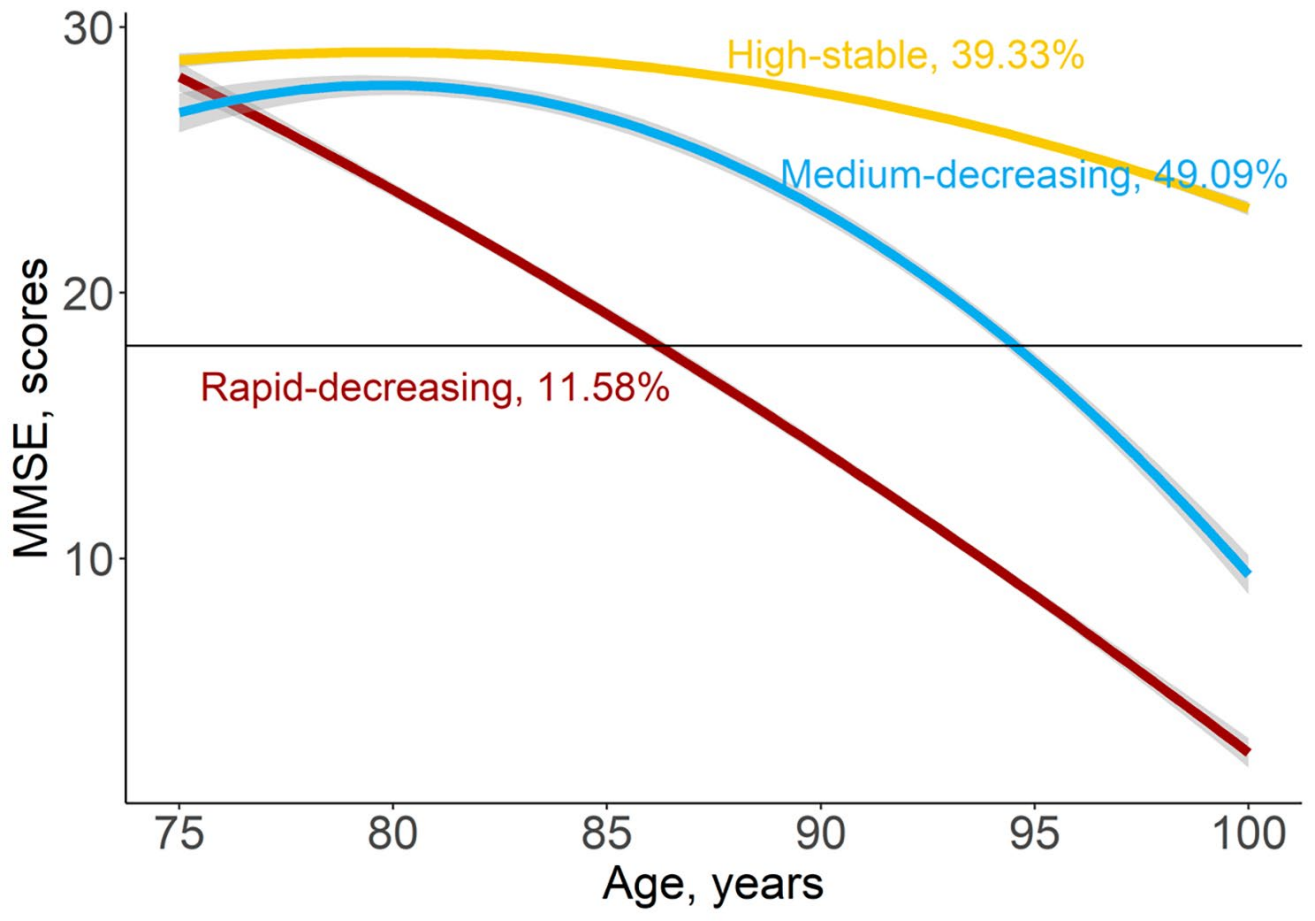
Predicted trajectories of cognitive function during 75–100 years old. The trajectories were shown in solid lines, and the 95% confidence intervals (CIs) were shown in shadow. The proportions in each trajectory were shown. (see detailed information on the curve parameters in Supplemental Table S3)

Figure 1 shows the longitudinal trajectories of MMSE during 75 ~ 100 years old of 3300 participants. Three distinct trajectories were established, labeled as high-stable (n = 1298, 39.33%), medium-decreasing (n = 1620, 49.09%), and rapid-decreasing (n = 382, 11.58%). In the high-stable group, MMSE persisted at a high level of 23 ~ 30 score. In the medium-decreasing group, MMSE decreased gradually from 28 score up to 10 score. In the rapid-decreasing group, MMSE decreased rapidly from 27 score up to 4 score.

After excluding respondents (n = 191) with arrhythmias, 3109 respondents were included in the subsequent analysis. Table 1 summarizes the baseline characteristics of study variables by MMSE trajectory classes. Compared with the high-stable group, the medium-decreasing group and the rapid-decreasing group were more likely single female, with higher proportion of village inhabitant, lower education level, lower proportion of smokers and alcohol drinkers, lower fruit intake, and lower proportion of PA subjects at baseline. Supplement Table S3 shows excluded respondents (n = 53,840) were generally older single females, with higher baseline body weight, lower proportion of smokers and alcohol drinkers, and lower proportion of PA subjects than those included (n = 3109).

**Table 1:** Characteristics at Baseline by Cognitive Function Trajectory Groups

Variable	High-stable	Medium-decreasing	Rapid-decreasing	*p* value
N	1226	1526	357	
Age, ys	81.8 (5.0)	81.6 (4.6)	80.0 (4.3)	< 0.001
Males, n (%)	913 (74.5)	400 (26.2)	88 (24.7)	< 0.001
Residence, n (%)				
City	322 (26.3)	341 (22.4)	58 (16.3)	
Town	388 (31.7)	491 (32.2)	107 (30.0)	
Village	516 (42.1)	694 (45.5)	192 (53.8)	< 0.001
Educational, n (%)	703 (57.3)	377 (24.7)	76 (21.3)	< 0.001
Marital, n (%)	653 (53.3)	495 (32.4)	126 (35.3)	< 0.001
Cohabitant, n (%)				
Family	1045 (85.2)	1188 (77.9)	278 (77.9)	
Solitary	149 (12.2)	281 (18.4)	59 (16.5)	
Live in institution	32 (2.6)	57 (3.7)	20 (5.6)	< 0.001
Smoker, n (%)	369 (30.1)	230 (15.1)	60 (16.8)	< 0.001
Drinker, n (%)	391 (31.9)	288 (18.9)	71 (19.9)	< 0.001
Fruit eater, n (%)	403 (32.9)	391 (25.6)	82 (23.0)	< 0.001
Vegetable eater, n (%)	1101 (89.9)	1321 (86.6)	299 (83.8)	0.002
PA, n (%)	558 (45.5)	493 (32.3)	92 (25.8)	< 0.001
Weight, kg	53.1 (9.8)	49.0 (9.2)	47.5 (8.3)	< 0.001
RHR, time/minute	73.0 [68.0, 79.0]	74.0 [69.0, 80.0]	72.5 [68.0, 78.0]	0.005
MMSE, score	28.0 (2.6)	25.9 (4.4)	22.2 (7.5)	< 0.001
Hypertension, n (%)	152 (12.4)	219 (14.4)	59 (16.5)	0.098
Diabetes, n (%)	14 (1.1)	24 (1.6)	3 (0.8)	0.432
Stroke, n (%)	37 (3.0)	39 (2.6)	23 (6.4)	< 0.001
Heart disease, n (%)	67 (5.5)	84 (5.5)	9 (2.5)	0.058

RHR, resting heart rate; PA, physical activity; MMSE, minimum mental state examination

According to the changing pattern of MMSE, individuals in high-stable group were defined as normal cognitive function. Those in medium/rapid-decreasing group were defined as cognitive decline. Table 2 presents odds radios (ORs) and 95% confidence intervals (CIs) of the association between RHR and cognitive function status. Compared with those whose RHR between 60 and 69 time/minutes, the ORs (95% CIs) of < 60, 70 ~ 79, 80 ~ 80, and > 90 groups were 1.11 (0.70, 1.79), 1.23 (1.07, 1.52), 1.40 (1.13, 1.73), and 1.51 (1.07, 2.16) in unadjusted model, respectively. After adjustment for baseline age, gender, residence, education level, marital status, cohabitant, smoking and alcohol drinking status, fruit and vegetable intake, self-report hypertension, self-report diabetes, self-report stroke, self-report heart disease, body weight, and PA, the ORs (95% CIs) were 1.19 (0.69, 2.05), 1.27 (1.03, 1.56), 1.30 (1.01, 1.67) and 1.62 (1.07, 2.47), respectively.

**Table 2:** ORs (95% CIs) of Resting Heart Rate for Cognition Trajectory Groups

RHR groups	Model 1^*^	Model 2^**^	Model 3^***^
< 60	1.11 (0.70, 1.79)	1.20 (0.70, 2.07)	1.19 (0.69, 2.05)
60 ~ 69	Reference	Reference	Reference
70 ~ 79	1.23 (1.07, 1.52)	1.25 (1.01, 1.53)	1.27 (1.03, 1.56)
80 ~ 89	1.40 (1.13, 1.73)	1.29 (1.01, 1.64)	1.30 (1.01, 1.67)
> 90	1.51 (1.07, 2.16)	1.58 (1.05, 2.39)	1.62 (1.07, 2.47)

OR, odds ratio; CI, confidence intervals; RHR,resting heart rate

* Unadjusted for any covariates

** Adjusted for baseline age and sex, residence, education level, marital status, cohabitant, smoking, and alcohol drinking

*** Adjusted for baseline age and sex, residence, education level, marital status, cohabitant, smoking and alcohol drinking, fruit eater, vegetable eater, physical activity, body weight, self-report hypertension, self-report diabetes, self-report stroke, and self-report heart disease

**Fig. 2:**
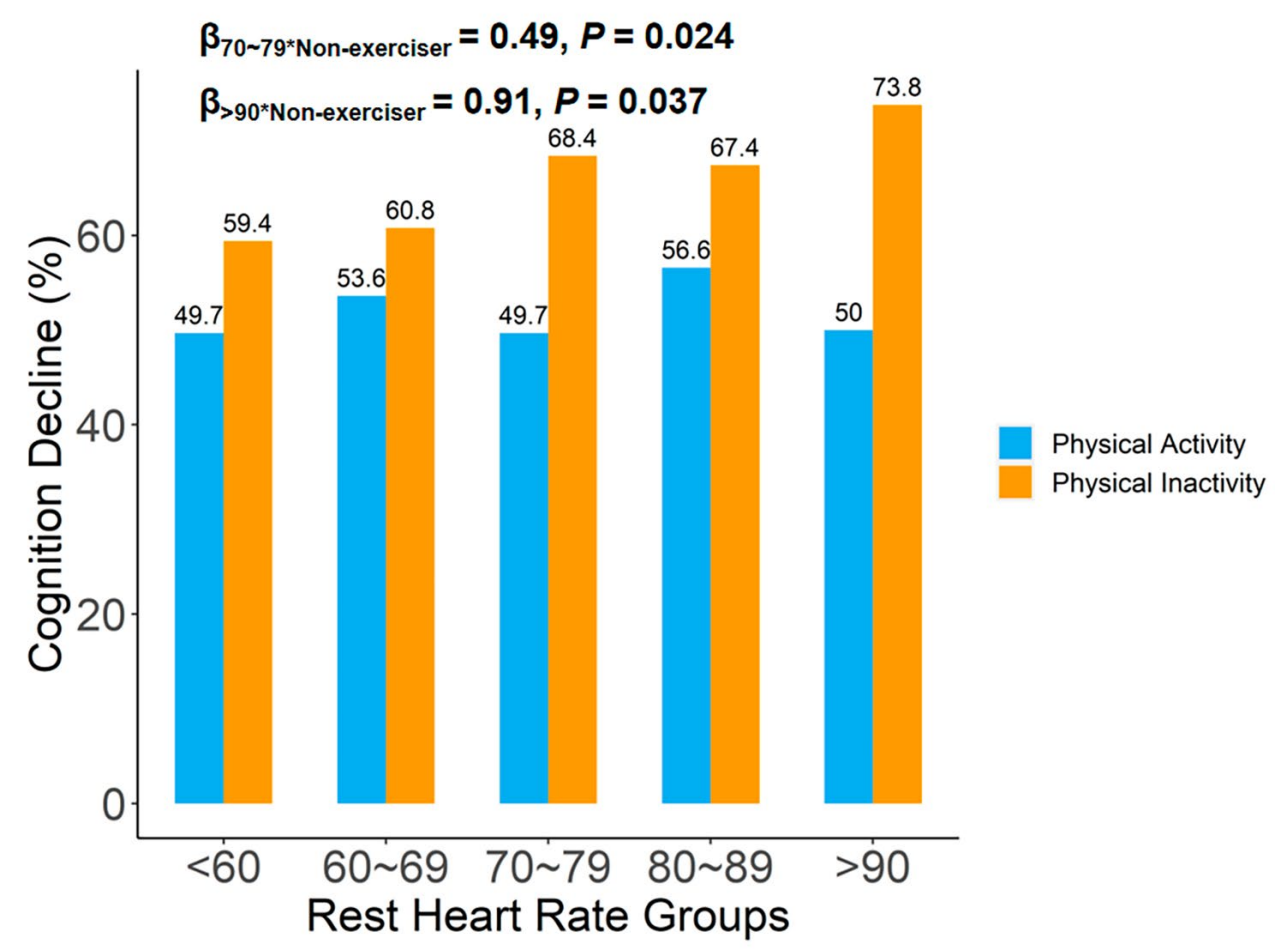
Rate of cognitive function decreasing in 75 ~ 100 years old, across different combined categories of RHR and physical activity status RHR, resting heart rate

The rate of cognitive function decreasing in 75 ~ 100 years old across different combined categories of RHR and PA status are presented in Fig. 2. The proportion of cognitive decline was higher in the physical inactivity groups than in the PA group with the same RHR, and an interaction effect on cognitive decline was found between RHR and PA (β_70~79*physical inactivity_ = 0.49, *P* = 0.024, β_>90*physical inactivity_ = 0.91, *P* = 0.037).

Table 3 presents ORs and 95% CIs of the difference of the association between RHR group membership and cognitive decline in PA subjects and physical inactivity subjects. After adjustment for baseline age, gender, residence, education level, marital status, cohabitant, smoking and alcohol drinking status, fruit and vegetable intake, self-report hypertension, self-report diabetes, self-report stroke, self-report heart disease, and body weight, the ORs (95% CIs) were 1.27 (0.51, 3.16), 0.97 (0.69, 1.35), 1.23 (0.82, 1.85), and 0.97 (0.49, 1.89) for < 60, 70 ~ 79, 80 ~ 80, and > 90 groups in PA subjects, respectively (*P* > 0.05 for all). While these association were 1.16 (0.59, 2.31), 1.56 (1.20, 2.05), 1.38 (1.01, 1.90) and 2.38 (1.38, 4.19) in physical inactivity individuals, respectively.

**Table 3:** ORs (95% CIs) of Resting Heart Rate for Cognition Trajectory Groups by Physical Activity Status

RHR groups	Physical activity *		Physical inactivity *	
	OR (95% CI)	*p* value	OR (95% CI)	*p* value
< 60	1.27 (0.51, 3.16)	0.600	1.16 (0.59, 2.31)	0.667
60 ~ 69	Reference	-	Reference	-
70 ~ 79	0.97 (0.69, 1.35)	0.837	1.56 (1.20, 2.05)	0.001
80 ~ 89	1.23 (0.82, 1.85)	0.328	1.38 (1.01, 1.90)	0.049
> 90	0.97 (0.49, 1.89)	0.922	2.38 (1.38, 4.19)	0.002

OR, odds ratio; CI, confidence intervals; RHR, resting heart rate

* Adjusted for baseline age and sex, residence, education level, marital status, cohabitant, smoking and alcohol drinking, fruit eater, vegetable eater, body weight, self-report hypertension, self-report diabetes, self-report stroke, and self-report heart disease

## Discussion

In the current study, we identified three distinct MMSE trajectories from 75 to 100 years in macrobian Chinese population, and found the higher RHR was a significant risk factor for the decreasing of cognitive functions. Although the normal range of RHR was recognized as 60 ~ 100 bpm, our results showed that the oldest old population should control the RHR at the range from 60 to 69 bpm to reduce the risk of cognitive decline. Furthermore, we found that the relationship between RHR and cognitive decline was modified by PA status, indicated macrobian individuals without PA may be considered as high-risk group to develop cognitive decline. Our observations supported that higher RHR would rise the risk of cognitive decline steeply, and this relationship was modified by PA. More attention should be paid to control RHR in macrobian Chinese population, especially in terms of those without PA, to prevent the decreasing of cognitive functions.

In this study, three distinct trajectories for MMSE during 75 ~ 100 years were identified. In the high-stable group, cognitive functions persisted at a high level of 26 ~ 30. In the medium-decreasing group, individuals presented a decreased cognition, with MMSE from 30 to 10. In the rapid-decreasing group, this trend of decline became steeply. Previous researches, using data from America, China, Japan, Korea, and Spain identified 2–4 distinct MMSE trajectories for old people [27–32]. Although the numbers of trajectory groups were different among these studies, they all found a high-stable group and a rapid-decreasing group, which were in line with us. Moreover, those researches usually focused on the early elderly life-course, such as 50 ~ 75 years old [31]. The literatures about the oldest old individuals were still limited. Our results illustrated that in the oldest old participants, the changing patterns of cognitive functions existed heterogeneity, widely. Some studies reported that the heterogeneity could be explained by some pathologic changes [30, 33]. However, some modifiable factors would also influence the speed of cognitive decline, even though adjustment those pathologic measurements [30, 31, 34]. This indicated that cognitive decline should not be regarded as a normal consequence of aging and neuropathological changes, and the modifiable factors can contribute to varying degrees of cognitive decline.

In this study, we discovered a more than 70 bpm RHR would rise the risk of cognitive decline, compared with those whose RHR was 60 ~ 69 bpm. A few studies have explored the relationship between RHR and cognitive decline [6, 12–15, 35, 36]. However, the results were controversial. Imahori, et al. demonstrated that compared with those RHR 60 ~ 69 bpm, individuals with more than 80 bpm showed a significant hazard ratio (HR) of cognitive decline (HR and 95% CI was 1.55 [1.06, 2.66]) [6]. Another research, based on the Atherosclerosis Risk in Communities (ARIC), was reported the similar results [13]. These results were consistent with our observations [6, 13]. Inversely, Wod, et al. illustrated that RHR may not associate with cognitive function in general population using data from Danish [14]. However, to our knowledge, this research only used limited measurements, ignoring the longitudinal changing patterns of cognitive function [14]. In our study, we found the level of MMSE were similar in baseline within three distinct trajectory groups, but the linear slope varied widely. Some individuals with steep trend of decline may be misclassified because of the short duration of follow-up (medium-decreasing group). European and Chinese guidelines for hypertension have listed high RHR (> 80 bpm) as an independent risk factor [37]. Nevertheless, whether this criterion was suitable for the oldest old people was still unknown. Our results supported the oldest old individuals should control RHR ranging from 60 to 69 bpm, and the interventions which could control RHR may have effect of preventing cognitive decline.

In this current study, we found the association of RHR and cognitive decline was modified by PA. Higher RHR would only rise the risk of cognitive decline in those without PA. Previous researches have explored the relationship between RHR, PA, and cognitive functions [6, 38]. Zabetian-Targhi, et al. has reported that PA would improvement the cognitive functions in people with type 2 diabetes [38]. Imahori, et al. demonstrated that individuals with RHR more than 80 bpm presented a significantly higher risk of the cognitive decline, compared with those RHR 60–69 bpm [6]. These results were all in line with the current study [6, 38]. However, previous researches all focused on the relationship between PA or RHR and cognitive functions separately [6, 38], ignoring the interaction between PA and RHR on the cognition. Our results supported that PA may play a modifier in RHR-cognitive functions association, and individuals with higher RHR but never exercising may be the high-risk sub-group of cognitive decline.

There are few studies on RHR and cognitive decline, but the relationship between high RHR and cognitive dysfunction seems reasonable. Firstly, high RHR, as a risk factor for cardiovascular and cerebrovascular events in the general population and high-risk population, may result in reverse hypertension, atrial fibrillation and heart failure, which are also vital risk factors for dementia [7, 39–41]. Secondly, high RHR is a sign of sympathetic hyperactivity [42]. Indeed, sympathetic overactivity may contribute to CVD (ventricular arrhythmias and myocardial ischemic events) and non-CVD (obesity, dyslipidemia, etc.) [40, 43], which are risk factors for cognitive decline and dementia. Thirdly, high RHR is related to the incidence and progression of vascular oxidative stress, endothelial dysfunction and atherosclerosis [44, 45]. The hemodynamic changes caused by the above factors may cause cognitive impairment through vasculitis change, cerebral circulation insufficiency, microthrombosis and other ways [7]. Besides, some studies reveal that reducing RHR can not only inhibit the formation of atherosclerotic plaques, but also promote the growth of vascular collateral circulation and protect vascular endothelium, which provides a possible basis for controlling heart rate and delaying the decline of cognitive function [46–48].

There are some strengths in the current study. Firstly, with large sample size and repeated measurements, CLHLS is a community-based longitudinal study. It allows us to identify the distinct trajectory groups of cognitive functions. Secondly, we discovered the association of RHR and cognitive decline was modified by PA, which could provide evidences for identifying the high-risk sub-groups of cognitive decline in oldest old people. Moreover, our study focused on the oldest old individuals, whose aged over 75 years old. To our knowledge, this is the first study to explore the relationship between RHR and cognition decline in Chinese community individuals.

On the other hand, some limitations should be acknowledged. Firstly, the definition of cardiovascular diseases was relying on self-reported data, which means we could not identify those with subclinical diseases. It may influence the relationship between RHR and cognitive decline. Secondly, although we adjusted covariates as we can in our models, unobserved confounders might still influence the results. For instance, information about BMI of participants were not available because the height was not collected in the first four follow-up. We could only adjust the body weight in the logistic models to reduce the bias caused by BMI. Thirdly, the information about physical activity was collected by questionnaire, and the information bias may exist. Besides, the data of exercise intensity and exercise duration were unavailable in the current study, further researchers should explore the relationship of RHR, PA, and cognitive decline based on more detailed data about PA. Finally, the CLHLS was a survey concentrating on Chinese old population. Our results may not be generalizable to other ethnic population.

## Conclusion

In conclusion, compared to those with RHR 60 ~ 69 bpm, individuals with a more than 70 bpm RHR presented a higher risk of cognitive decline rapidly, and this association was modified by PA. Our findings provide evidence that elder people with higher RHR but never exercise may be the high-risk subgroup of cognitive decline. Public health intervention for controlling RHR and encouraging PA during the oldest old life-course has important significance on the prevention of cognitive decline.

### Electronic supplementary material

Below is the link to the electronic supplementary material.

Supplementary Material 1

## Data Availability

Data availability statement Data are available in a public, open access repository from The Chinese Longitudinal Healthy Longevity Survey (https://opendata.pku.edu.cn/dataverse/CHADS).
