# Supplementary material for: Associations between resting heart rate and cognitive decline in Chinese oldest old individuals: a longitudinal cohort study
Source: BMC Geriatr. 2024 Jan 4;24:14. doi: 10.1186/s12877-023-04600-y (PMC10768207; doi:10.1186/s12877-023-04600-y)
Supplement: Supplementary file 1 — Supplementary Material 1 [file 12877_2023_4600_MOESM1_ESM.docx]

**Supplemental Materials**

**Table S1**. Latent Class Growth Mixture Models (LCGMM) results of model fitting process for cognitive function

| No. Latent class | Polynomial degree | Log-Lik | BIC | % Participants per class | Mean posterior probabilities | % Posterior probabilities > 70% |
| --- | --- | --- | --- | --- | --- | --- |
| 1 | Linear | -67594 | 135228 | 100 | na | na |
|  | Quadratic | -67541 | 135140 | 100 | na | na |
|  | Cubic | -67541 | 135145 | 100 | na | na |
| 2 | Linear | -67370 | 134813 | 45.70/54.30 | 0.83/0.85 | 76.86/80.36 |
|  | Quadratic | -67292 | 134683 | 40.64/59.36 | 0.83/0.87 | 76.44/82.80 |
|  | Cubic | -67273 | 134669 | 59.36/40.64 | 0.87/0.83 | 83.10/77.93 |
| 3 | Linear | -67311 | 134728 | 11.58/51.03/37.39 | 0.79/0.77/0.82 | 67.54/68.53/75.53 |
|  | **Quadratic** | **-67225** | **134588** | **11.58/49.09/39.33** | **0.79/0.76/0.83** | **69.37/68.15/76.35** |
|  | Cubic | -67224 | 134618 | 35.91/40.30/23.79 | 0.79/0.62/0.66 | 67.76/25.49/31.85 |
| 4 | Linear | -67291 | 134720 | 39.00/11.39/36.67/12.94 | 0.79/0.77/0.79/0.69 | 74.98/63.83/68.51/45.20 |
|  | Quadratic | -67188 | 134555 | 18.42/30.61/37.52/13.45 | 0.75/0.69/0.74/0.66 | 59.54/40.30/64.94/39.41 |
|  | Cubic | -67210 | 134640 | 25.33/6.52/35.24/32.91 | 0.60/0.58/0.71/0.66 | 21.89/19.53/50.04/39.87 |

No. Latent class: latent class number of the model; Log-Lik: the maximum Log-Likelihood; BIC: the Bayesian information Criterion; % Participants per class: proportion of participants per class; The best fitting model is highlighted in bold characters. (NA: not applicable).

**Table S2**. Parameters estimates for the best fitting 3-class quadratic Latent Class Growth Mixture Model for cognitive function

|  |  | Intercept (se)* | Linear (se) | Quadratic (se) |
| --- | --- | --- | --- | --- |
| **Fixed effect** | | | | |
|  | Rapid-decreasing | 33.726 (1.9505) | -2.618 (0.1472) | 0.069 (0.0238) |
|  | Medium-decreasing | 59.092 (1.4755) | -2.184 (0.0894) | -0.073 (0.0128) |
|  | High-stable | 77.833 (0.6932) | -1.118 (0.0827) | -0.084 (0.0118) |
| **Random effects: variance-covariance matrix** | | | | |
| σ^2^_int_ = 0.289 | | | | |
| σ^2^_linear slope_ = 0.677 | | | | |
| σ^2^_quadratic slope_ = 0.002 | | | | |
| σ^2^_error_ = 20.88 | | | | |

se = standard error

*: Intercept interpreted as the expected level of cognitive function in scores at 86 years of age (centering to the mean age of the sample).

**Table S3.** Baseline and follow-up characteristics of participants included and excluded

| Variable | Included | Excluded | *p* value |
| --- | --- | --- | --- |
| N | 3109 | 53840 |  |
| Age, ys | 81.5 (4.7) | 88.0 (11.9) | <0.001 |
| Males, n (%) | 1401 (45.1) | 22228 (41.3) | <0.001 |
| Residence, n (%) |  |  |  |
| City | 721 (23.2) | 13909 (25.8) |  |
| Town | 986 (31.7) | 15697 (29.2) |  |
| Village | 1402 (45.1) | 24234 (45.0) | <0.001 |
| Educational, n (%) | 1156 (37.2) | 17903 (35.2) | 0.022 |
| Marital, n (%) | 1274 (41.0) | 16169 (30.1) | <0.001 |
| Cohabitant, n (%) |  |  |  |
| Family | 2511 (80.8) | 44499 (82.9) |  |
| Solitary | 489 (15.7) | 7169 (13.4) |  |
| Live in institution | 109 (3.5) | 2032 (3.8) | 0.002 |
| Smoker, n (%) | 659 (21.2) | 9084 (16.9) | <0.001 |
| Drinker, n (%) | 750 (24.1) | 9843 (18.4) | <0.001 |
| Fruit eater, n (%) | 876 (28.2) | 18663 (34.7) | <0.001 |
| Vegetable eater, n (%) | 2721 (87.6) | 45383 (84.4) | <0.001 |
| PA, n (%) | 1143 (36.8) | 14749 (27.5) | <0.001 |
| Weight, kg | 50.0 [45.0, 55.0] | 49.0 [41.0, 60.0] | 0.014 |
| RHR, time/minute | 74.0 [68.0, 80.0] | 74.0 [68.0, 80.0] | <0.001 |
| Hypertension, n (%) | 430 (13.8) | 10666 (20.2) | <0.001 |
| Diabetes, n (%) | 41 (1.3) | 1940 (3.7) | <0.001 |
| Stroke, n (%) | 99 (3.2) | 3120 (6.0) | <0.001 |
| Heart disease, n (%) | 160 (5.2) | 5062 (9,7) | <0.001 |

RHR, resting heart rate; PA, physical activity.
